# Pubertal gynecomastia incidence among 530,000 boys: a cross sectional population based study

**DOI:** 10.3389/fped.2024.1367550

**Published:** 2024-03-06

**Authors:** Ori Berger, Tzipi Hornik-Lurie, Ran Talisman

**Affiliations:** ^1^ Plastic Surgery Unit, Barzilai University Hospital Medical Center, Ashkelon, Israel; ^2^ Department of Data Research at the Research Authority, Meir Medical Center, Kfar Saba, Israel

**Keywords:** gynecomastia, plastic surgery, adolescents, incidence, big data

## Abstract

**Background:**

Adolescent gynecomastia, a benign proliferation of male breast tissue, can lead to psychological issues during adolescence. The prevalence varies widely (4%−69%). The incidence peaks are during neonatal, pubertal, and senescent periods. Its affect on emotional well-being necessitates understanding and occasional intervention. This study aimed to determine the incidence of gynecomastia among male adolescents aged 12–15 years.

**Methods:**

A retrospective cross-sectional study utilized the Clalit Health Care Services database (2008–2021) with a population of approximately 4.5 million. Participants aged 12–15 years were included if diagnosed with gynecomastia (International classification of diseases-9 code 611.1) and having a body mass index (BMI) measurement and no obesity diagnosis (ICD9 code 278.0). Data analysis included incidence rates and associations with ethnicity, age, BMI, and socioeconomic status.

**Results:**

531,686 participants included with an incidence of 1.08%. Of all participants, 478,140 had a BMI ≤ 25 with an incidence of 0.7%, and 0.25%–0.35% yearly, and 70% of gynecomastia patients were aged 13–14 years. The prevalence of gynecomastia differed between Jews (1.28%) and Arabs (0.67%), but the disparity diminished when socioeconomic status was considered.

**Conclusions:**

This unprecedented Population study establishes a definitive rate of true pubertal gynecomastia, revealing a lower yearly incidence as compared to previous reports. The higher observed prevalence among Jewish adolescents, may be caused due to complex interactions between different influencing factors. Understanding these dynamics can aid in formulating more targeted interventions and policy strategies to address gynecomastia's affect on adolescent well-being.

## 1 Introduction

Adolescent gynecomastia, characterized by benign glandular proliferation in the male breast resulting in enlargement (1–5), can lead to embarrassment and emotional distress among adolescents, negatively impacting self-esteem, psychological well-being, and social development, potentially resulting in psychological disorders (1, 2, 6).

While several hypotheses have been proposed, the exact cause remains unclear (2, 7). Pubertal gynecomastia typically arises from transient imbalances between androgens and estrogens, with over 95% of cases being considered physiological (7). It's essential to differentiate true gynecomastia from pseudo-gynecomastia, characterized by fat accumulation in the breast without glandular proliferation, often observed in obese males (5). Pathological causes of gynecomastia in adolescents are rare, accounting for less than 5% of cases and stemming from various pathological conditions (7).

Gynecomastia exhibits three peaks of incidence throughout life: neonatal, pubertal, and senescence (1, 2). Pubertal gynecomastia can manifest as early as age 10, typically occurring around 13–14 years of age or at Tanner stage 3–4 (1, 5). Although gynecomastia is usually asymptomatic, it may cause pain and tenderness in the mammary gland. In most cases, physiological gynecomastia resolves spontaneously within 1–3 years, requiring only careful monitoring and reassurance. However, severe cases or those causing significant psychological distress may warrant intervention. Surgery may be considered after a minimum observation period of 12 months for persistent enlargement, intractable pain or tenderness, and significant psychosocial distress (7).

The reported prevalence of adolescent gynecomastia varies widely, ranging from 4% to 69%, leading to controversy and conflicting findings in past studies (1–5). Accurately defining the incidence is crucial for understanding the condition's nature, improving clinicians' approach, and facilitating timely and appropriate interventions and mitigate secondary emotional implications.

Based on the senior author clinical experience, our study hypothesizes that the incidence of gynecomastia among male adolescents aged 12–15 years falls towards the lower end of the above reported spectrum. Notably, this study boasts an unprecedentedly large sample size drawn from the Clalit Health Care Service (CHS) database, Israel's largest health maintenance organization. Our objectives include assessing the condition's incidence among male adolescents aged 12–15 and investigating potential associations with ethnicity.

## 2 Methods

In our retrospective cross-sectional study, we harnessed expansive data resources of the CHS's, which covers over 4.5 million enrollees and runs a network of 14 hospitals and over 1,300 primary clinics. We utilized data from the entire population. Data were collected from the years 2008–2021. Due to the observational nature of the study with no identifiable information used, participants were not required to provide informed consent. The ethics approval was obtained from the CHS's institutional ethics committee in accordance with the Helsinki Declaration, on 15 March 2023.

The study included male patients aged 12–15 years, diagnosed with gynecomastia as identified using the International Classification of Diseases (ICD-9) code 611.1 “hypertrophy of breast”, with a body mass index (BMI) measurement. Females and files without BMI measurement were excluded.

Individuals were defined as having gynecomastia if this diagnosis was documented at least once in their electronic record by community physicians as outpatients.

Data collection encompassed newly diagnosed cases of gynecomastia, alongside information on sex, age, ethnicity, BMI, obesity (ICD-9 code 278.0), and socioeconomic status. All extracted from the CHS database in 2023. Sex and ethnicity were ascertained from Israeli identity documents issued by the Ministry of Interior.

The primary objective was to determine the incidence of gynecomastia. A patient was considered to have gynecomastia if they received an ICD-9 code 611 diagnosis at any point between the ages of 12 and 15 years during the study period, the typical onset period. Diagnoses of gynecomastia were established based on either patient or guardian complaints or observations made by pediatricians during routine physical examinations.

The exposure in the study was ethnicity, categorized as either Jewish or Arab, determined based on the place of residence. A limitation of this method is that communities with an Arab majority were classified as Arab, and those with a Jewish majority were classified as Jewish. This limitation arises from the way data is collected and stored in Clalit's database. BMI, Primary care physician's (PCP) office visits, and socioeconomic status were confounding factors. BMI was classified as either ≤25 or >25. The ICD-9 code 278.0 for obesity was also considered a confounding factor. Socioeconomic status was defined in the database as low, medium, or high according to Israel's Central Bureau of Statistics based on income. All were obtained from the CHS database.

The primary objective of the study was to determine the incidence of gynecomastia in male patients aged 12–15 years. Secondary objectives included exploring possible associations with age and ethnicity within the same group of participants.

Categorical data were presented through frequency and percentage distributions, while continuous variables were summarized by means and standard deviation. BMI, a continuous variable, was categorized into: ≤ 25 (indicative of normal weight), or >25, aligned with the World Health Organization's definition of overweight, in an attempt to distinguish excess body fat (pseudo-gynecomastia) from glandular proliferation in the male breast (true gynecomastia). This categorization enables a focused examination of the true incidence of gynecomastia among male adolescents with normal weight, mitigating the influence of obesity-related breast enlargement. Age was stratified into four categories: 12, 13, 14, and 15 years.

The primary outcome, the incidence of gynecomastia diagnoses was analyzed using chi square test. Secondary outcomes, exploring associations between gynecomastia and BMI, age, ethnicity, and socioeconomic status were analyzed using chi-square tests. To compare the average number of PCP visits between ethnicity subgroups *T*-Test analysis was used. For socioeconomic status one-way ANOVA was used. A multivariant analysis using hierarchical binary logistic regression was performed to determine the relationship between ethnicity and socioeconomic status on gynecomastia development. To control the confounding factor of weight, we focused on the cohort of subjects with BMI ≤ 25 and no diagnosis of ICD-9 278.0 for obesity. Statistical analyses were executed using SPSS/PC statistical software, version 26.0, with the significance level set at *p* < 0.001.

## 3 Results

**Figure 1:**
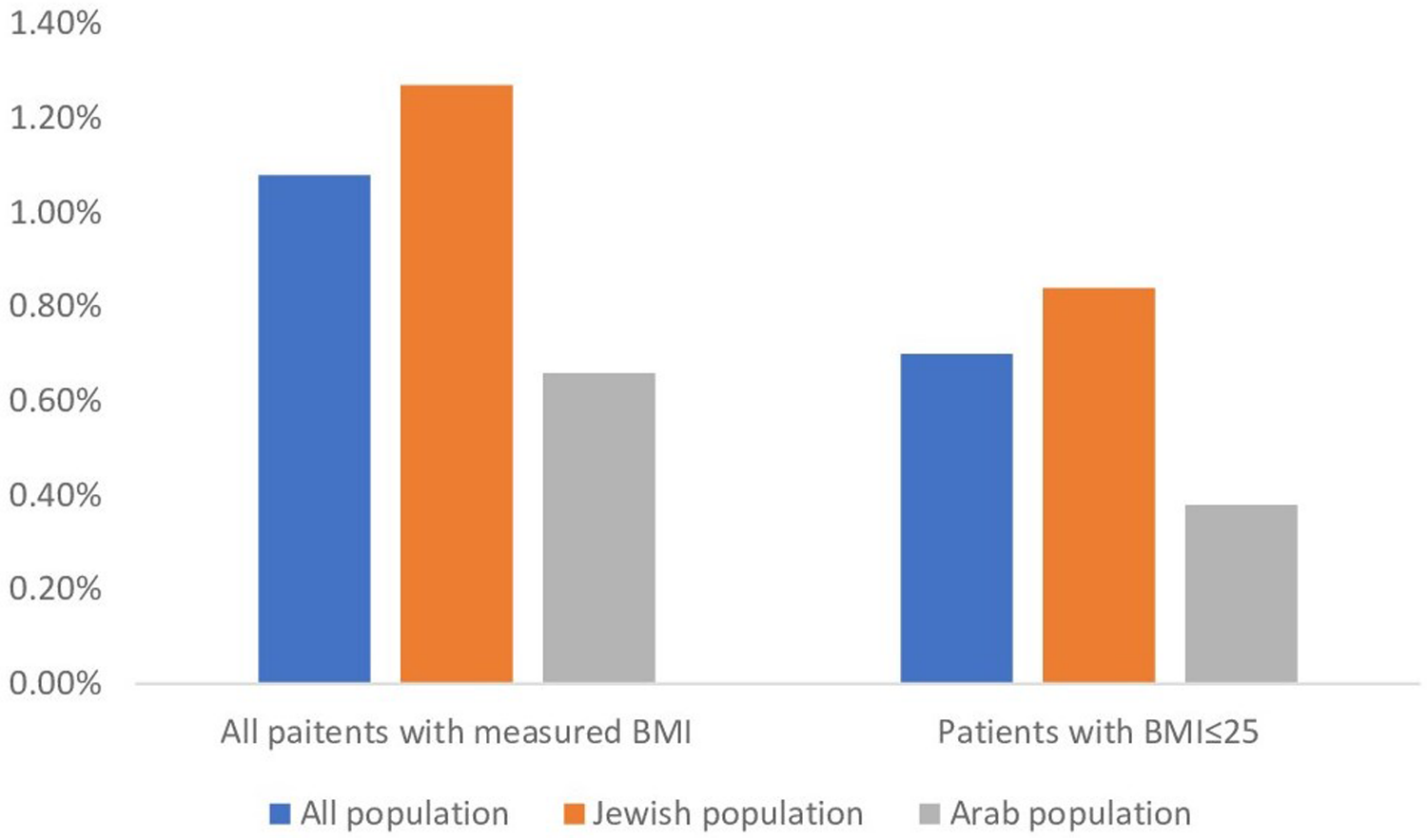
Gynecomastia rates nin All BMI and BMI ≤ 25 per ethnicity. The figure shows the incidence rates of gynecomastia in the total population and in those with BMI ≤ 25 by for the general population and by ethnicity group.

In this cross-sectional study spanning 2008–2021, 596,184 adolescents aged 12–15 were analyzed, revealing only 6,458 (1.08%) of them with gynecomastia. With 478,140 (80.2%) cases presenting with a BMI ≤ 25 and no diagnosis of obesity. Among those with gynecomastia, 4,167 (70%) had a BMI ≤ 25, resulting in an estimated true gynecomastia incidence of 0.70% in the general adolescent population (Table 1, Figure 1). The age of incidence distribution showed that 16.3% of cases occurred in 12-year-olds, 38.5% in 13-year-olds, 32.7% in 14-year-olds, and 12.4% in 15-year-olds, with the mean age at diagnosis calculated at 13.3 years (±0.92) and a 95% confidence interval ranging 11.52–15.08 years.

**Table 1:** Study subjects’ characteristics.

	General	Arab	Jewish	Significance
BMI ≤ 25 (*N*, %)	478,140 (80·2)	143,829 (89·41)	334,311 (90·15)	*p* < 0·001
Gynecomastia (*N*, %)	6,458 (1·08)	1,192 (0·66)	5,266 (1·27)	*p* < 0·001
BMI ≤ 25 & Gynecomastia (*N*, %)	4,167 (0·7)	678 (0·38)	3,489 (0·84)	*p* < 0·001
Total (*N*)	596,184	179,908	416,276	–

BMI, body mass index.

Ethnicity played a role in gynecomastia incidence, with the study including 30.3% of children identifying as Arabs and 69.7% as Jews, aligning with the ethnic composition in Israel. Among these, 0.67% of Arab children and 1.28% of Jewish children were diagnosed with gynecomastia, as demonstrated in Table 1. Chi-square test underscored a substantial association between ethnicity and gynecomastia occurrence (*χ*^2^ = 425.53 and *p* < 0.001). Additionally, among patients with gynecomastia in the Arab group, 56.9% had a BMI of ≤25, while in the Jewish group, 66.3% had a BMI of ≤25. This association between ethnicity and gynecomastia occurrence was also statistically significant (*χ*^2^ = 40.26, *p* < 0.001).

**Figure 2:**
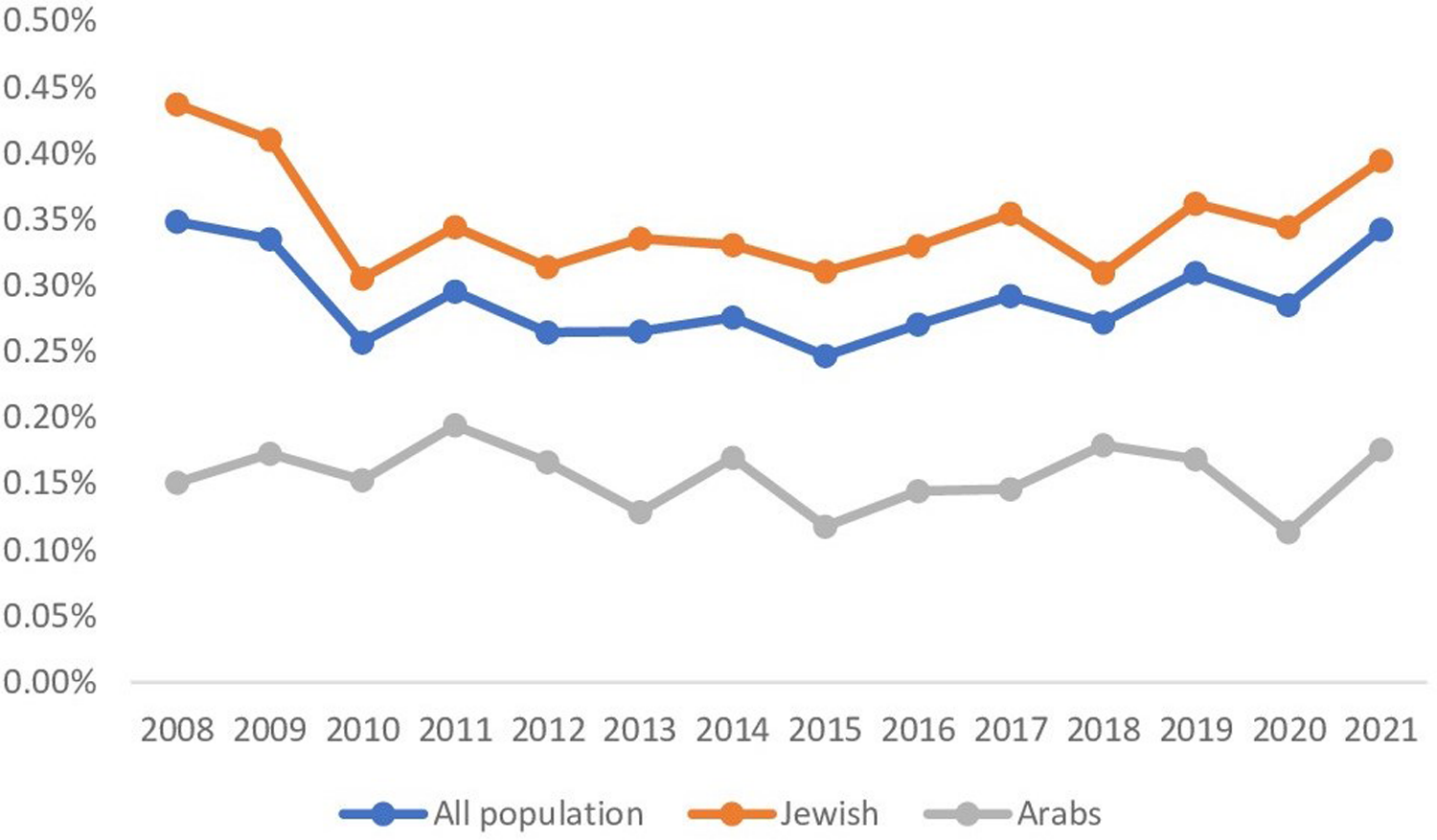
Gynecomastia trends over the years 2008–2021. The figure shows gynecomastia trends over time for patients with BMI ≤ 25 divided among the general (blue), Jewish (orange), and Arab (grey) populations. The rates are consistent for all groups.

In the years 2008–2021, an annual enrollment of 124,251–154,033 children was insured in CHS, with most of them, 55,393–112,957, having a BMI ≤ 25. Among those, 193–383 (0.25%–0.35%) cases of gynecomastia with BMI ≤ 25 diagnosed annually (Figure 2). The Jewish population enrolled in CHS included 38,184–85,154 children per year with BMI ≤ 25 and an incidence of gynecomastia of 167–336 (0.31%–0.44%) cases, also indicating a steady ratio throughout the years. The annual enrollment of the Arab population in CHS ranged 17,209–37,357 children with BMI ≤ 25, and a yearly incidence of 26–63 gynecomastia cases (0.11%–0.19%) (Figure 2).

The data revealed differences in the mean number of visits to a PCP over the years 2008–2021. In the general population with a BMI ≤25, the mean number of visits was 11.23 (±10.68), compared to 16.31 (±12.3) in the gynecomastia group with a BMI ≤25. Within the Arab group with BMI ≤ 25, the mean number of PCP visits was 8.65 (±9.23) per patient. In contrast, in the Jewish group with BMI ≤ 25, the mean number of PCP visits was 12.34 (±11.06) per patient. Statistical tests, employing two-sided analyses, identified significant differences in PCP visit rates between the Arab and Jewish groups across all BMI categories (*p* < 0.001), as shown in Table 2.

**Table 2:** Mean number of PCP visits per patient during study.

	Arab	Jewish	Significance	Total
All population [*N*]	8·26 (±9·15) [179,908]	11·63 (±11·04) [416,276]	*p* < 0·001	10·61 (±10·62) [596,184]
BMI ≤ 25 [*N*]	8·65 (±9·23) [143,829]	12·34 (±11·06) [334,311]	*p* < 0·001	11·23 (±10·68) [478,140]
Gynecomastia [*N*]	14·31 (±11·25) [1,192]	17·2 (±13·16) [5,266]	*p* < 0·001	16·66 (±12·87) [6,458]
BMI ≤ 25 & Gynecomastia [*N*]	13·87 (±10·38) [876]	16·82 (±12·6) [4,208]	*p* < 0·001	16·31 (±12·3) [5,084]

BMI, body mass index; PCP, primary care physician.

Socioeconomic status also played a role in the findings, with Arabs generally having a lower socioeconomic status (69%) compared to Jews (17%, respectively, *χ*^2^ = 147,842, *p* < 0.001). Patients with gynecomastia were associated with a higher socioeconomic status (23%) than the general population (15%), which was statistically significant (*p* < 0.001). Furthermore, patients with a high socioeconomic status tended to have more PCP visits (12.21 ± 11.03) compared to those with medium (11.35 ± 10.85) or low (8.9 ± 9.77) socioeconomic status (*p* < 0.001 based on a two-sided test), as shown in Table 3.

**Table 3:** Socioeconomic status and subjects characteristics.

	Low status	Medium status	High status	Significance
All population (*N*, %)	179,975 (32)	292,657 (53)	82,922 (15)	–
BMI ≤ 25 (*N*, %)	143,444 (32)	233,436 (52)	68,825 (15)	–
BMI ≤ 25 & Gynecomastia (*N*, %)	852 (18)	2,722 (57)	1,236 (25)	–
Ethnicity				*p* < 0·001
- Arab (*N*, %) - Jewish (*N*, %)	112,275 (69) 67,700 (17)	49,569 (30) 243,088 (62)	1,124 (1) 81,798 (21)	
PCP visits (mean)	8·9 (±9·77)	11·35 (±10·85)	12·21 (±11·03)	*p* < 0·001

BMI, body mass index; PCP, primary care physician.

**Figure 3:**
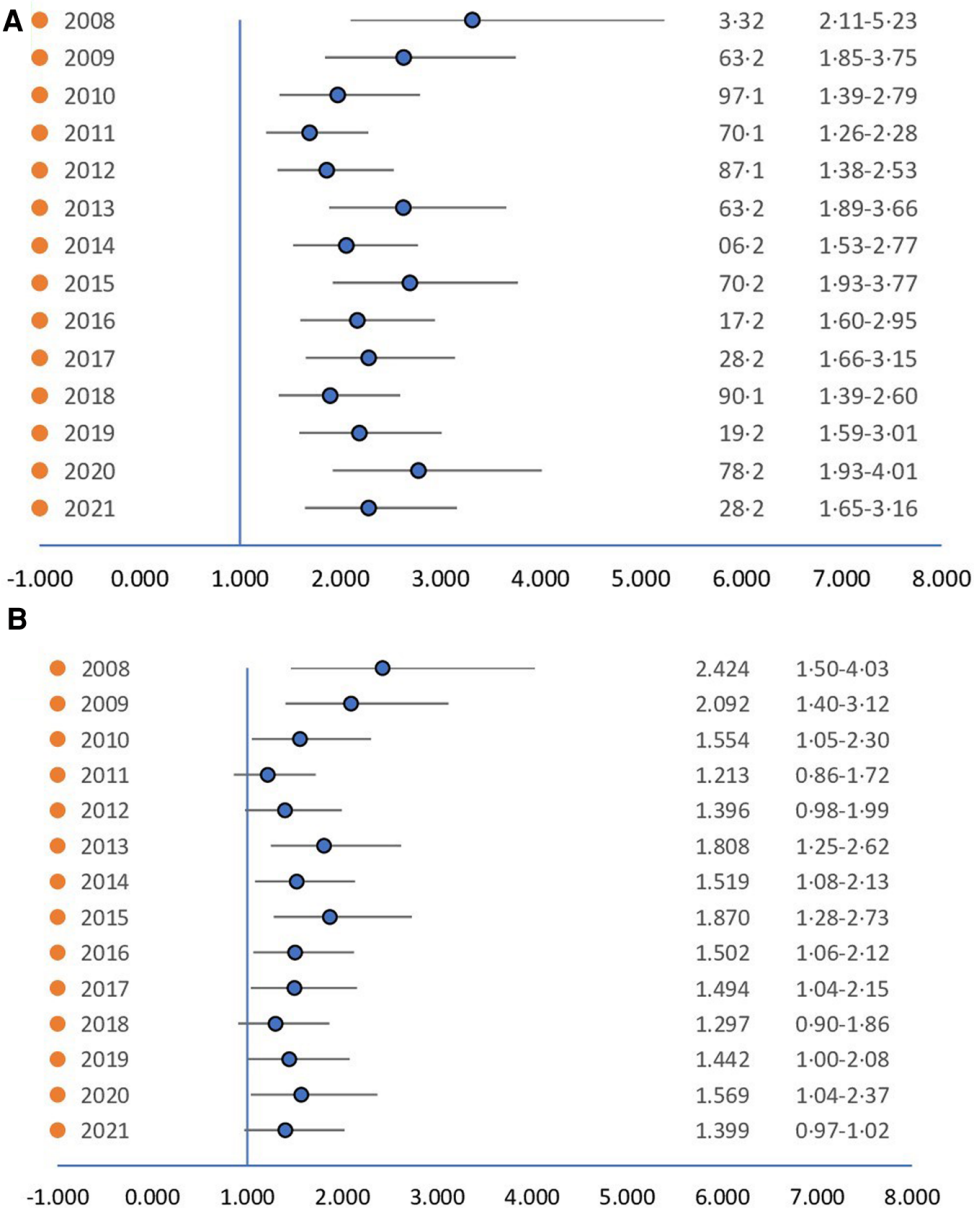
(**A**) Hazard ratio of ethnicity. (**B**) Hazard ratio of ethnicity controlling for socioeconomic status. When controlling for socioeconomic status the difference loses from its significance.

To explore the affect of socioeconomic status on the ethnicity's influence on incidence, a logistic regression analysis was conducted on individuals with a BMI ≤ 25 (Figure 3). When accounting only for ethnicity (Figure 3A) the differences between the ethnic groups remained significant. However, when accounting for both ethnicity and socioeconomic status (Figure 3B) the differences lessened their significance, highlighting the complex interplay between ethnicity and socioeconomic factors in the incidence of gynecomastia among adolescents.

## 4 Discussion

In our study, we noted an occurrence rate of gynecomastia to be 1.08%, with a 0.7% occurrence rate among individuals with BMI ≤ 25. These percentages are lower than prevalence rates documented in certain prior studies (2, 4, 8) (Table 4) and deviate from the elevated prevalence rates outlined in earlier research (3–5, 8). This marked necessitates additional deliberation. The most accurate approach to assess the incidence of pubertal gynecomastia among young adults would involve deploying physicians skilled in physically examining the condition in public schools. Examinations would be conducted on all males aged 11–16 years. This process would facilitate the identification of young males with a normal BMI and clear clinical indicators of genuine gynecomastia, thereby establishing the precise incidence. Unfortunately, conducting such a study is impossible in our country due to strict constraints by the Ministry of Education. To ascertain the genuine occurrence of gynecomastia, we employed a relatively novel measurement approach: a vast database that enabled precise analysis, akin to the methodology employed in the Danish study conducted by Koch et al. (15). The principal aim of our study was to evaluate the frequency of gynecomastia amongst male adolescents aged 12–15 years, a period recognized for experiencing the highest incidence of this condition (1). In this age group, there are two primary forms of gynecomastia: true physiological gynecomastia and pseudo-gynecomastia, which is associated with excessive weight gain (5). We differentiated between these two forms by excluding cases with a diagnosis of obesity or a BMI above 25, as outlined in the methods section. The study cohort encompassed data from over 500,000 individuals in the years 2008–2021.

**Table 4:** Summary of previous publications.

Study	No. subjects	Criteria used	Age (years)	No (%) with gynecomastia	Other
Nydick (9)	1,865	0·5 ≥ cm	10–16	722 (38.71)	–
Neyzi (10)	993	Firm subareolar tissue	9–17	70 (7·05)	–
Lee (11)	29	Firm subareolar tissue	Pubertal boys	20 (68·97)	Required signed informed consent
Fara (12)	681	0·5 ≥ cm	11–14	228 (33·48)	Potential estrogen exposure
Harlan (13)	3,522	1 ≥ cm	12–17	147 (4·17)	–
Moore (14)	135	0·5 ≥ cm	8·5–17·5	30 (22·22)	–
Biro (3)	377	Palpable glandular tissue	10–15	183 (49·54)	Required signed informed consent
Koch (15)	204,618	Per ICD10 code	10–15	995 (0·49)	–
Kumanov (2)	3,082	1 ≥ cm	10–19	121 (3·93)	–
Total	215,302	–	–	2,516 (1·17)	–

ICD10, international classification of diseases 10.

One of the most cited articles on the prevalence of pubertal gynecomastia by Braunstein (4), along with other significant previous works, has reported rates of 35%–69% cases (3, 4, 9, 11, 12, 14, 16). Nydick (9) and Moore (14) defined gynecomastia based on a diagnostic criterion of a palpable breast disk of 0.5 centimeters or less. However, current recommendations advocate leaving at least a 1-centimeter disc of breast tissue under the areola following surgery to prevent depression of the nipple areolar complex (17). Furthermore, research has shown a strong correlation between the appearance of gynecomastia on CT scans and mammograms (18), leading to the suggestion that breast glandular tissue measuring less than 2 centimeters should be considered a normal finding (19). A diagnosis of gynecomastia should require the presence of breast glandular tissue measuring at least 2 centimeters (19–21). Nuttal (16), in his study, included patients across a broad age range of 17–58 years, which could potentially have influenced the research outcomes. Fara (12) speculated that estrogen exposure through food had an affect on gynecomastia rates in his study, while Lee's study (11) solely focused on participants who had provided signed parental informed consent, a factor that could introduce bias into the results. The underdiagnosis of the condition (20) due to fewer PCP examinations is a possible explanation for the lower incidence rates in this study, but the overall high rate of PCP appointments for subjects during this study period contradicts this. Other possible explanations for the variations from previous published data could be that, as mentioned, earlier studies failed to accurately define gynecomastia through physical examination (9, 21) or had other methodological flaws, such as biases in participant selection (12, 11), incorrect population sampling, or small study cohorts (14, 16, 11).

In contrast, more recent studies have published lower prevalence rates, ranging from 4% to 7% (19, 20) (Table 4). Kumanov et al. (2). conducted a study in 2007 involving 3,082 healthy males aged 10–19 years. They utilized a diagnostic criterion of a palpable button of firm subareolar breast tissue measuring at least 1 centimeter in diameter and found an incidence rate of 3.93% for pubertal gynecomastia. Similarly, Koch and colleagues (15) reported an incidence rate of 0.49% among 204,618 boys aged 10–15 years old. By aggregating the findings from all studies (2, 3, 9–15), the collective sample size comprised 215,302 subjects. These findings reveal an average gynecomastia rate of 1.17%, which better aligns with the rate found in our study among the general population (Table 4).

Upon further analysis of gynecomastia incidence among different ethnic groups, we observed that 1.28% of Jewish individuals and 0.67% of Arab individuals with BMI ≤ 25 were diagnosed with gynecomastia. Despite Jews and Arabs sharing a genetic origin, and no clear correlation between ethnic groups in past studies (3), the difference in incidence found in our study may stem from two factors. Arabs exhibited a lower socioeconomic status and a higher prevalence of increased BMI. This lower socioeconomic status was linked to reduced rates of gynecomastia diagnosis, fewer PCP visits, and lower medical expenditure, as highlighted in prior research (22–24). The likelihood of underdiagnosis and underreporting the condition among Arab participants during routine pediatrician visits is a well-known phenomenon in the Arab ethnicity (25). These various factors could collectively contribute to the observed differences in gynecomastia incidence between the two ethnicities, as supported by the logistic regression analysis preformed. This reinforces the hypothesis that the disparity is not primarily attributable to ethnic group differences.

Our study also unveiled significant variations in gynecomastia incidence across different age groups. The highest incidence was found among 13-year-olds, with an incidence of 38.5%, while the lowest incidence was observed among 15-year-olds, with an incidence of 12.4%. The mean age at the diagnosis of gynecomastia was 13.4 years, which aligns with the findings of previous studies (2–4, 9, 11, 13).

The strengths of our mega data study lie in its ability to examine data over a long period of time allowing for the examination of gynecomastia incidence trends over the years, providing insights into changes over time. This research also includes a large group of adolescents, over 500,000 subjects, which enhances the statistical power and generalizability of the results. This provides consistent incidence rates of gynecomastia throughout the study's duration. This consistency was observed across the various subgroups (BMI, ethnicity). This holds intriguing implications for our understanding of factors contributing to gynecomastia during puberty.

This study has limitations due to its retrospective nature and is based on a computerized big database rather than a true population-based study, potentially introducing biases from misclassified information. Gynecomastia diagnosis may vary among different healthcare providers, affecting the accuracy of the results. Overdiagnosis may inflate the reported incidence, while underdiagnosis may underestimate it. The results from this database are aligned with previous studies (2, 15). Furthermore, the CHS database undergoes continuous validation processes (26). The principle of volume aggregation also applies here, as errors tend to be essentially random, thereby offsetting and canceling each other as more data is incorporated (27). Thus, we assert that this study offers the most precise estimation achievable of the incidence of true pubertal gynecomastia and serves as the best possible proxy for a study population.

Misclassification of ethnicity and BMI poses potential limitations. In the attempt to isolate true gynecomastia, BMI was restricted to ≤25, however true gynecomastia may be present in higher BMI. Ethnicity classification into Arab and Jewish groups might oversimplify Israel's diverse population. As ethnicity is determined in CHS by the subject's municipality of residence, mixed cities are counted as Jewish due to the Jewish majority in them. This possible misclassification may affect any perceived association between gynecomastia and ethnicity found in this article. Mitigating such a bias in such a large number of people is challenging as the personal information is coded and crossing the information with another large database such as Israel's national identity database is not feasible. BMI is a flawed indicator of obesity because it doesn't consider variations in body composition. There are alternatives for the BMI index such as body fat percentage, Dual-Energy x-ray Absorptiometry, to name a few. While these examinations may be more accurate, they were not available in the CHS database. Therefore, BMI serves as a reasonable but imperfect measure for our research that may have caused some misclassification bias.

The study raises concerns about underdiagnosis and underreporting while shedding light on potential healthcare disparities between groups. Although this topic has been previously explored in publications (22–25), future research can delve into its correlation with cultural norms and body image perceptions among diverse societal groups.

This research addresses the incidence of pubertal gynecomastia, in contrast to prevalence in other studies. Consequently, making a comparison between the different publications is more challenging. We contend that, given the predominantly temporary nature of gynecomastia, incidence serves as a more suitable indicator for defining the epidemiology of this condition.

As the integration of electronic records into healthcare systems is indispensable for clinical-care and research, the validity of record accuracy and completeness is important. The emergence of large databases has provided an unparalleled solution to a longstanding clinical debate, which previously could only be resolved through extensive long-term prospective studies, if they were feasible to conduct. In this study, population data, as opposed to a sample, were leveraged to access a cohort three times larger than the combined total of all previous publications (2, 3, 9–15), thus providing a more precise answer to the question of pubertal gynecomastia incidence.

## 5 Conclusions

This retrospective cross-sectional study has unveiled a notably lower incidence of gynecomastia in the general population, estimated at 1.08%, with a prevalence of 0.7% among individuals with a BMI ≤ 25 as compared to earlier reports. Among those diagnosed with gynecomastia, the highest frequency was among 13-year-olds (38.3%). This investigation has shed light on disparities in gynecomastia incidence between Jewish and Arab ethnic groups, with a higher incidence among Jewish participants. This disparity appears to be influenced by factors such as socioeconomic status, BMI, and potentially other variables. By harnessing a large database and the law of large numbers, this study has provided to date an unprecedented estimation of the rate of pubertal gynecomastia. Thus, it has significantly advanced our comprehension of this condition, offering valuable insights into its incidence, and contributing factors.

## Data Availability

The data analyzed in this study is subject to the following licenses/restrictions: Per request from Clalit health services. Requests to access these datasets should be directed to the Helsinki committee (meirhelsinki@clalit.org.il).
